# Recent establishment of tick-borne encephalitis foci with distinct viral lineages in the Helsinki area, Finland

**DOI:** 10.1080/22221751.2019.1612279

**Published:** 2019-05-14

**Authors:** Teemu Smura, Elina Tonteri, Anu Jääskeläinen, Gabriel von Troil, Suvi Kuivanen, Otso Huitu, Lauri Kareinen, Joni Uusitalo, Ruut Uusitalo, Tuula Hannila-Handelberg, Liina Voutilainen, Simo Nikkari, Tarja Sironen, Jussi Sane, Janne Castrén, Olli Vapalahti

**Affiliations:** a Department of Virology, University of Helsinki, Helsinki, Finland; b Division of Clinical Microbiology, Helsinki University Hospital Laboratory Services (HUSLAB), Helsinki, Finland; c Centers for Military Medicine and Biothreat Preparedness, Helsinki, Finland; d Archipelago Doctors Ltd, Parainen, Finland; e Natural Resources Institute Finland (Luke), Helsinki, Finland; f Department of Geosciences and Geography, University of Helsinki, Helsinki, Finland; g Department of Veterinary Biosciences, University of Helsinki, Helsinki, Finland; h Department of Health Security, Infectious Disease Control and Vaccinations Unit, National Institute for Health and Welfare, Helsinki, Finland

**Keywords:** Tick-borne encephalitis, flavivirus, incidence, phylogeny, Finland

## Abstract

Number of tick-borne encephalitis (TBE) cases has increased and new foci have emerged in Finland during the last decade. We evaluated risk for locally acquired TBE in the capital region inhabited by 1.2 million people. We screened ticks and small mammals from probable places of TBE virus (TBEV) transmission and places without reported circulation. The TBEV positive samples were sequenced and subjected to phylogenetic analysis. Within the study period 2007–2017, there was a clear increase of both all TBE cases and locally acquired cases in the Helsinki area. The surveillance of ticks and small mammals for TBEV confirmed four distinct TBEV foci in the Helsinki area. All detected TBEV strains were of the European subtype. TBEV genome sequences indicated that distinct TBEV lineages circulate in each focus. Molecular clock analysis suggested that the virus lineages were introduced to these foci decades ago. In conclusion, TBE has emerged in the mainland of Helsinki area during the last decade, with at least four distinct virus lineages independently introduced into the region previously. Although the overall annual TBE incidence is below the threshold for recommending general vaccinations, the situation requires further surveillance to detect and prevent possible further emergence of local TBE clusters.

## Introduction

Tick-borne encephalitis virus (TBEV) is a flavivirus (genus Flavivirus, family Flaviviridae) that causes severe encephalitis across large parts of Europe and Northern Asia. Globally, TBEV has been estimated to annually cause 13,000 cases of central nervous system infection [1]. In nature, TBEV circulates in a fragile cycle involving ticks and their vertebrate hosts, mainly small mammals. Humans are accidental hosts and do not contribute to the circulation of TBEV.

There are three subtypes of TBEV: European (Eur), Siberian (Sib) and Far-Eastern (FE). TBEV-Eur is carried mainly by *Ixodes ricinus* ticks in central and north-eastern Europe, whereas TBEV-Sib and -FE are found mainly in Ixodes persulcatus ticks in an area reaching from north-eastern Europe to the Russian Far East, China and Japan [1]. Recently, new subtypes of TBEV (Himalayan and Baikalian) have been characterized [2,3].

**Figure 1.**
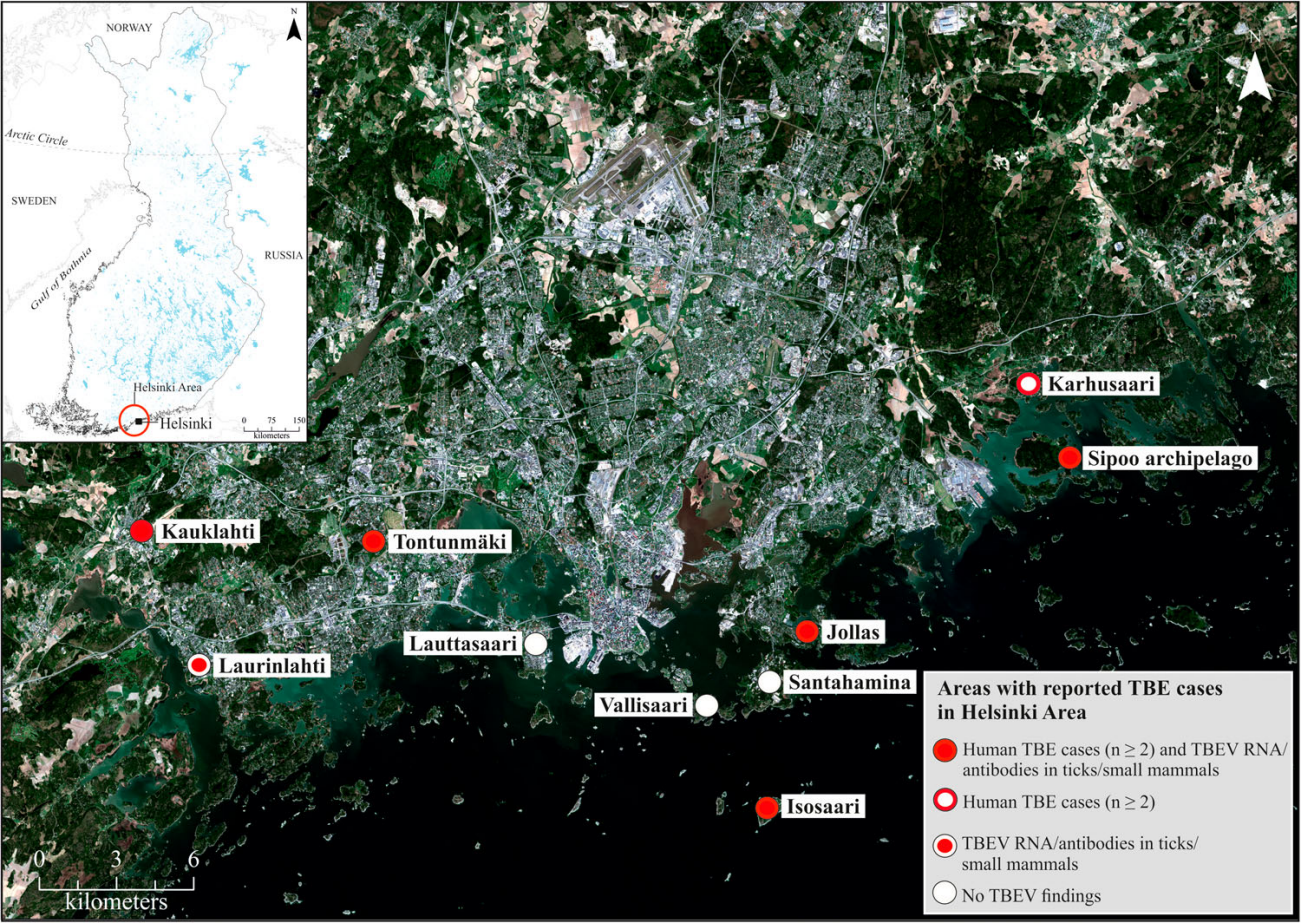
Map of collection sites.

The number of tick-borne encephalitis (TBE) cases has increased during the last decade in Finland. Finland lies at the northernmost limit of the TBE endemic area in Europe, and the occurrence of TBE in Finland is currently restricted to geographically separate endemic foci [4–7]. In the 1960s, screening of cattle for anti-TBEV antibodies demonstrated the circulation of TBEV in a few individual locations: the archipelagos of Åland and Turku, and the Lappeenranta and Kokkola regions [8]. TBEV transmission was restricted to these locations for decades until Isosaari Island in the outer archipelago of Helsinki was recognized as a TBEV focus in the 1990s (Figure 1) [9]. Since 2008, new TBE foci have been detected annually in Finland [4,10–12].

Apart from Isosaari Island, TBEV was not considered to circulate in the Helsinki area prior to 2011, when the first human case with a confirmed local transmission in Helsinki was reported. Since then, new locally acquired human TBE cases have occurred every year in the Helsinki region. The distribution of TBEV is considered to be affected by climate, mainly through its impact on the vectors and their hosts. The apparent increase in the locally acquired TBE case numbers is compatible with the establishment of new TBEV foci in southern Norway [13–17] and in the lake district of Sweden [18–20].

The recent increase of TBE cases in the densely populated and rapidly growing capital area, home to approximately 1.2 million inhabitants, together with the estimations of increased microclimatic suitability for the establishment of TBEV foci in the southern coast of Finland [21], are of concern and lead us to conduct the current study of TBEV in the area. We investigated the number and origin of TBE cases reported in the Helsinki area to detect locally acquired TBEV infections. To affirm local transmission, we collected ticks and small mammals within the Helsinki area, and screened them for TBEV. Virus genomes were sequenced and subjected to phylogenetic analysis that revealed a pattern of multiple introductions of distinct TBEV lineages to the Helsinki region.

## Material and methods

### Human cases

TBE is a notifiable disease in Finland and the cases are reported in the National Infectious Diseases Register (NIDR) of the National Institute for Health and Welfare (THL) (https://thl.fi/ttr/gen/rpt/tilastot.html). The register data of each acute case is studied and, if possible, the patients are interviewed to determine the most likely site of infection. The method has been described earlier [10]. Only cases with the known most likely site of infection were included in the primary epidemiological analyses.

### Study area

The Helsinki Metropolitan area covers the cities of Espoo, Helsinki, Kauniainen and Vantaa. In addition, the Helsinki area covers also the municipalities of Kirkkonummi, Vihti, Nurmijärvi, Tuusula, Kerava, Järvenpää, Hyvinkää and Sipoo. The population of the Helsinki area was 1,214,447 inhabitants at the end of 2017 (https://www.tilastokeskus.fi/tup/tilastotietokannat/index.html). All TBE cases during the years 2007–2017, in which any one of the municipalities of the Helsinki area was registered as the place of infection, place of diagnosis or as the home municipality of the patient, were included in this study. The TBE incidence trends were studied using joinpoint regression model implemented in Joinpoint Trend Analysis Software [22].

### Sample collection

Ticks, rodents and shrews were collected from various locations in Helsinki, Espoo and the Sipoo archipelago between 2013 and 2017 (Figure 1 and Table 1). The collection was focused on the probable sites of infection of the TBE cases. Tick collections were conducted by flagging questing ticks. Small mammals were collected using snap traps set over night and either dissected immediately or stored at −80 °C until dissected. Tick samples were also acquired from a crowdsourced collection initiative organized by a private medical clinic that provides TBEV vaccination in the area. This collection was not targeted towards suspected TBEV endemic foci.

**Table 1.** Tick and mammal sampling sites, years and TBEV-RT-PCR and TBEV-IFA results.

Site, year	Human cases	Ticks TBEV RNA pos/total	Mammal species (n)	Brains TBEV RNA pos/total	Spleens TBEV RNA pos/total	Serology TBEV Ab pos/total
Sipoo archipelago, 2013	1 in 2011 2 in 2016	5/90	*Myodes glareolus* (21)	0/21	0/20	1/21
			*Microtus agrestis* (1)	0/1	0/1	0/1
			*Apodemus flavicollis* (3)	0/3	0/3	0/3
			*Sorex* sp (2)	0/1	0/2	0/2
Helsinki Karhusaari, 2013	1 in 2016	0/9	*Myodes glareolus* (1)	N/A	N/A	0/1
			*Apodemus flavicollis* (1)	0/1	0/1	0/1
Helsinki Jollas, 2016–18	2 in 2014 1 in 2017	0/62	*Myodes glareolus* (26)	1/26	0/26	6/26
			*Microtus agrestis* (4)	0/4	0/4	0/4
			*Apodemus flavicollis* (3)	0/2	0/3	0/3
			*Sorex araneus* (2)	0/2	0/2	0/2
Helsinki Santahamina, 2015–2017	no cases	0/18	*Myodes glareolus* (30)	0/27	0/29	0/30
			*Microtus agrestis* (7)	0/7	0/7	0/7
			*Apodemus flavicollis* (8)	0/8	0/8	0/8
			*Sorex* sp (3)	0/2	0/3	0/3
Helsinki Vallisaari, 2016	no cases	0/34	No samples			
Helsinki Lauttasaari 2016–2017	no cases	0/80	No samples			
Espoo Tontunmäki 2014, 2016	1 in 2013	0/6	*Myodes glareolus* (6)	N/A	N/A	0/6
			*Microtus agrestis* (2)	N/A	N/A	0/2
			*Apodemus flavicollis* (8)	N/A	N/A	0/8
Espoo and Kauniainen 2016–2017	see Figure 1	14/679				

### Nucleic acid extraction and TBEV-RT-PCR

The ticks and tissue samples (brain and spleen) were homogenized in Dulbecco’s PBS + 0,2% BSA and sterile sand using a MagnaPure homogeniser at 7000 rpm for 2 × 30 s. RNA was extracted using TriPure reagent according to the manufacturer’s instructions (Roche) or AllPrep DNA/RNA Mini Kit (Qiagen) and eluted in dH2O. TBEV RT-PCR was performed as described previously [23], using Taqman fast virus 1-step mastermix (Thermo Fischer Scientific), 500 nM primers and 200 nM probe.

### Virus isolation

Homogenized ticks were suspended in PBS, inoculated to SK-N-SH human neuroblastoma cells and incubated for 1 h at +37°C. Tick suspension was replaced by Dulbecco’s Modified Eagle’s Media (DMEM) supplemented with glutamine, 100 units/ml penicillin, 100 μg/ml streptomycin and 2% foetal bovine serum (FBS). Samples were collected immediately after infection and at day 6 for RNA quantification and the cells were prepared for immunostaining on day of collection (6 dpi). RNA was extracted from the cell culture supernatant using the Viral RNA Mini Kit (Qiagen) according to the manufacturer’s instructions.

### Sequencing

The primers for complete TBEV coding region sequencing were designed using PrimalScheme tool http://primal.zibraproject.org/ [24] using 500 bp target amplicon size and 50 bp overlap. cDNA was sythesized from RT-PCR positive RNA samples with SuperScript III enzyme (Invitrogen) using random hexamers, and PCR was conducted using Q5 PCR kit (New England Biolabs) under previously published conditions [24]. The PCR products were purified using AmpureXP magnetic beads (Beckman Coulter) and sequencing libraries prepared using Nextera XT kit (Illumina) according to the manufacturer’s instructions.

The TBEV RT-PCR positive vole brain was homogenized at 3000 rpm for 60 s. Prior to RNA extraction, the homogenate was treated with a cocktail of micrococcal nuclease (New England BioLabs, Ipswich, USA) and benzonase (Millipore) for 1 h at +37°C. Ribosomal RNA was removed using a NEBNext rRNA depletion kit (New England BioLabs), according to the manufacturer’s protocol. The sequencing library was prepared using a NEBNext Ultra II RNA library prep kit (New England BioLabs).

The library fragment sizes were measured using agarose gel electrophoresis and the concentrations using Qubit Broad-Range dsDNA Assay Kit (Life Technologies) and NEBNext Library Quant kit for Illumina (New England BioLabs). Sequencing was conducted using MiSeq V2 reagent kit with 150 bp reads.

Raw sequence reads were trimmed and low quality (quality score <30) and short (<50 nt) sequences removed using Trimmomatic [25]. The trimmed sequence reads were de-novo assembled using Velvet de novo assembler [26] followed by re-assembly against the de-novo assembled consensus sequences using BWA-MEM algorithm [27] implemented in SAMTools version 1.8 [28].

In case no products were obtained using complete coding sequence PCR, amplification of smaller fragments of E-gene and NS5 were attempted using previously described protocols [29].

### Phylogenetic analysis

Complete genome and E gene sequences of all available European subtype TBEV were downloaded from GenBank (accessed June 2018). The sequences were aligned using the ClustalW algorithm followed by manual refinement. Substitution model was estimated using MEGA7 [30].

Phylogenetic tree based on the complete coding sequences was constructed using Bayesian Monte Carlo Markov Chain (MCMC) method implemented in BEAST version 1.8.0 [31]. Two different clock models (strict clock and log normal relaxed clock) and two demographic models (constant and Bayesian skyline models) were compared by calculation of Bayes Factors (ratio of marginal likelihoods of the models). The marginal likelihoods of different model combinations were estimated using path sampling and stepping stone methods [32,33]. Bayes factors (BF) were calculated for each pair of models. The final analysis was conducted using General Time Reversible (GTR) substitution model with 4-category gamma-distributed variation among sites and a proportion of invariant sites, strict clock model and Bayesian skyline demographic model. The analyses were run in duplicates for 50 million states and sampled every 5000 steps. Posterior probabilities were calculated with a burn-in of 10% and checked for convergence using the Tracer version 1.7. [34].

Phylogenetic tree based on E gene was inferred using the Bayesian method implemented in MrBayes v3.1.2 [35], using the GTR model, a 4-category gamma-distribution model of among-site rate heterogeneity and a proportion of invariant sites. MrBayes was run for 5 million generations, with final standard deviations between 2 runs of 0.011.

The analyses were carried out on the CSC server (IT Center for Science Ltd., Espoo, Finland).

### Immunofluorescence assay

The small mammal samples were screened for the presence of TBEV antibodies using an immunofluorescence assay on TBEV-infected cells on 10-well micro-titer well slides and detected using FITC-conjugated mouse immunoglobulins secondary antibody (Agilent), as described previously [36].

## Results

### TBE cases

**Figure 2.**
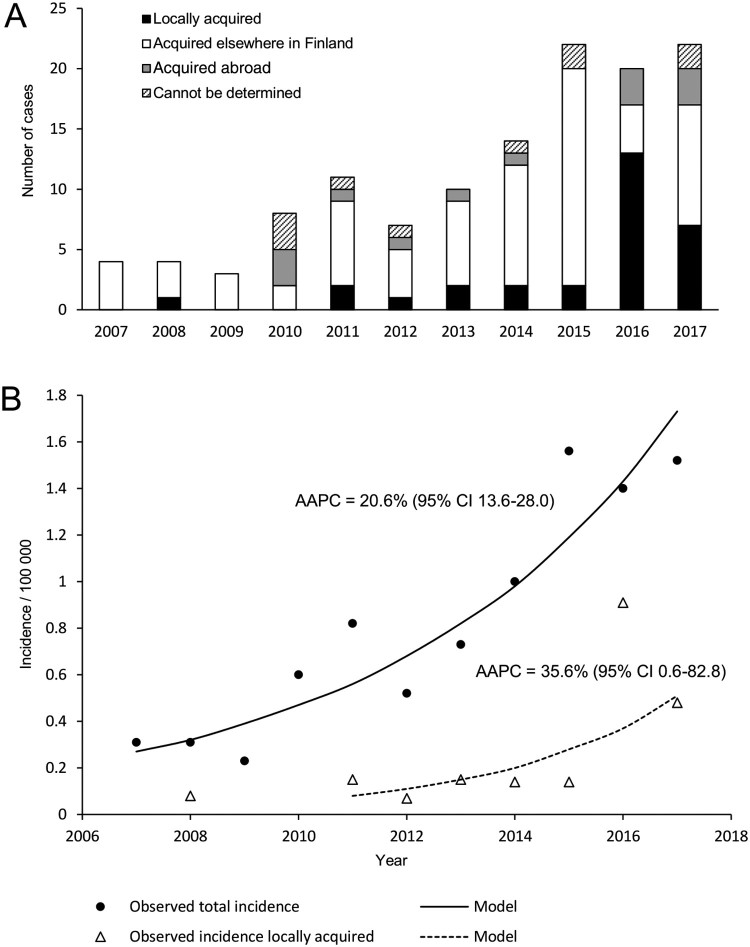
The number of TBE cases (A) and the TBE incidence (B) in the Helsinki area.

During the years 2007–2017, altogether 488 TBE cases were registered in the National Infectious Diseases Register (NIDR) of Finland. Out of these, 125 cases were diagnosed from the residents of the Helsinki area (Figure 2). The most likely location at which the tick bite was acquired was determined from an interview of each TBE patient by National Institute for Health and Welfare (NIHW), as described previously [10]. In 35 cases, the reported site of infection was in the Helsinki area. Thirty of these were residents of the Helsinki area. Eighty-five infections had most likely been acquired during visits to other known endemic foci in Finland (*N* = 72) or abroad (*N* = 13). In ten cases the probable location of infection could not be determined. Within the study period of 2007–2017, there was a clear increase in both the annual total number of TBE cases (from 4 cases in 2007 to 22 cases in 2017) and locally acquired TBE cases (from 0 to 13 locally acquired cases in 2016 and 7 locally acquired cases in 2017) in the Helsinki area (Figure 2).

The incidence of all TBEV cases reported in the Helsinki area increased from 0.31 to 1.52/year/100,000 inhabitants [average annual percentage change (AAPC) 20.6% (95% confidence interval 13.6–28.0; *P* < .05)], and the incidence of locally acquired TBEV infections increased from zero to 0.48/year/100,000 inhabitants (AAPC 35.6% [CI 0.6–82.8; *P* < .05]).

### Screening of ticks and small mammals

Five out of 90 ticks collected in 2013 from an island in Sipoo archipelago were TBEV RNA positive, and one bank vole (Myodes glareolus) was TBEV antibody positive (Table 1). One patient had contracted TBEV most likely on this island in 2011. From the Jollas peninsula in Helsinki, where two patients in 2014 and one patient in 2017 had likely contracted the disease, one out of 35 bank voles was TBEV RNA positive, and six had antibodies against TBEV.

Altogether 679 ticks were collected from humans, pets or directly from the vegetation by volunteer citizens from various locations in Espoo and Helsinki in 2016–2017. Of these ticks, 15.3% were engorged. Fourteen ticks (2.1%) were TBEV RNA positive. Twelve of the TBEV positive ticks were collected from two pet cats (five and seven positive ticks, respectively) in Espoonkartano/Kauklahti, which was also a likely site of infection for one TBE patient in the year 2017.

The other two locations where TBEV positive ticks were detected were Tontunmäki, where three cases of TBE have been detected previously, and Laurinlahti, where no human cases have been detected.

### Molecular epidemiology

Sequence data were obtained from twelve RT-PCR positive ticks and one bank vole brain. In addition, the complete genome of a previously detected positive tick pool from the Isosaari island (year 2005) [37] was sequenced. All TBEV strains were of the European subtype (Figure 3). The sequences obtained from each given district of the Helsinki area showed local geographic clustering, but the strains from the different districts did not share a common ancestor. The strains from Espoo formed a monophyletic group that clustered together with the strains from Kuutsalo, Kotka, which is located approximately 140 km from Espoo [38] and Bornholm Denmark [39]. Furthermore, this group formed a sister clade to strains detected in Switzerland [40]. The strains from Sipoo formed another monophyletic group. Strains from Latvia [41] and Estonia [42] formed an outgroup for this cluster. The strain sequenced from bank vole brain (Jollas) clustered together with strains from Latvia [41]. The TBEV strain from Isosaari clustered together with strains from Slovenia [43], Czech Republic [44] and Germany [45], as previously reported [37]. None of the newly detected strains from Espoo, Sipoo or Jollas grouped together with the previously detected TBEV strain from Isosaari, Helsinki, which comprised the fourth TBEV lineage within the Helsinki area. Figure 3.Maximum clade credibility tree constructed from complete coding sequences (A) and E genes (B).

**Figure:**
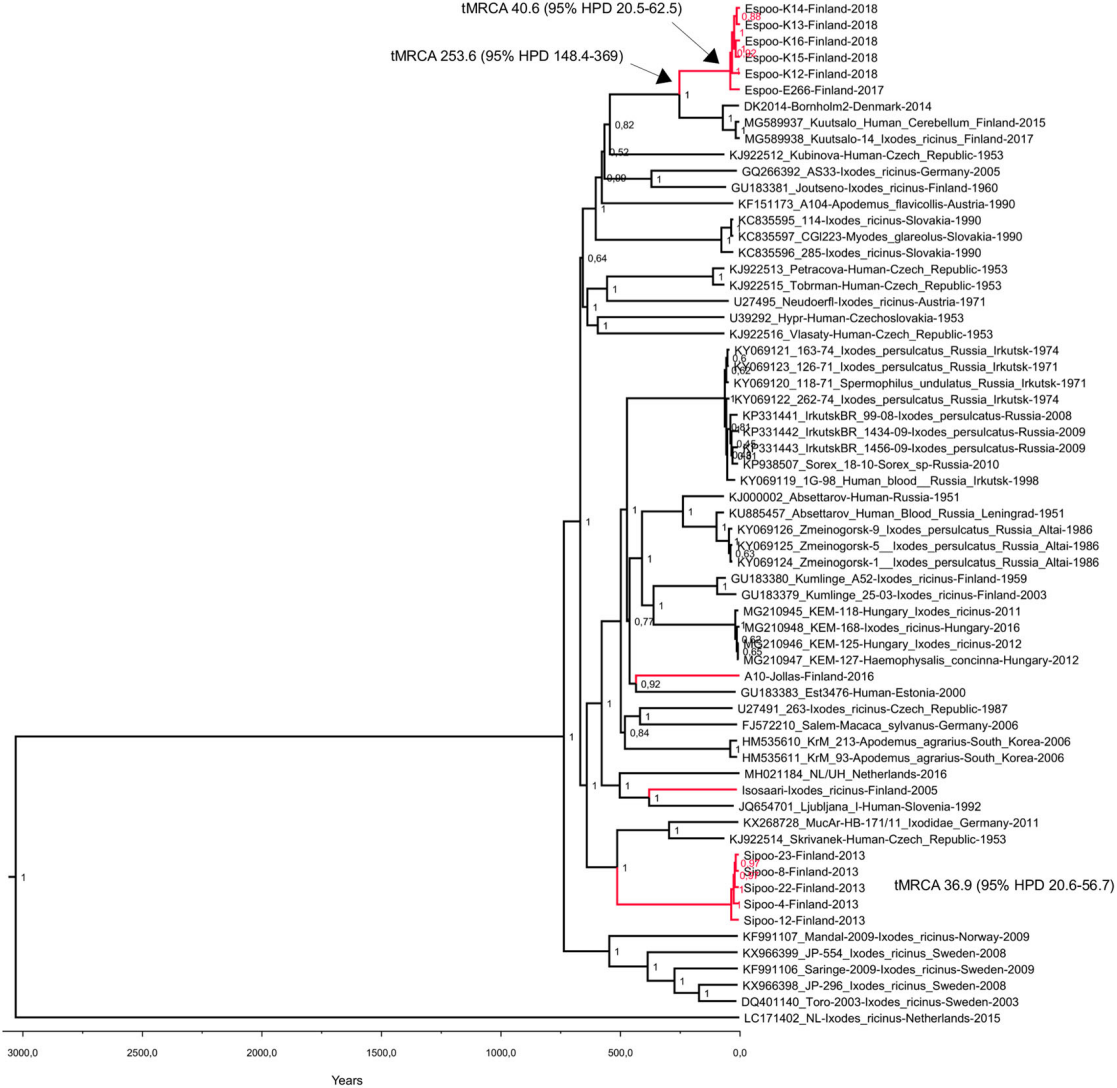

**Figure:**
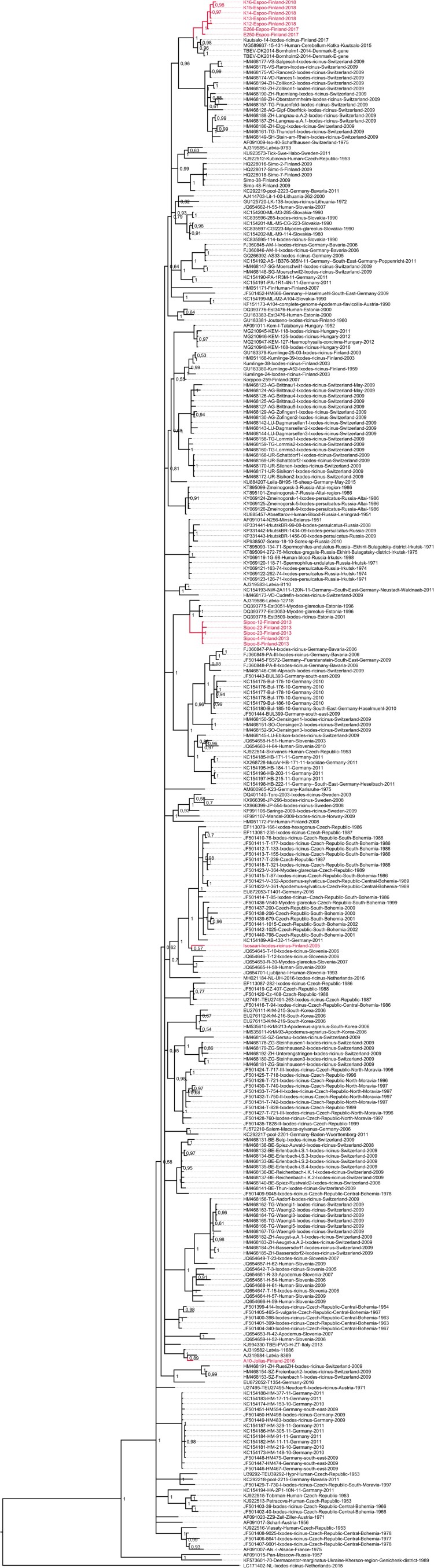

The pairwise genetic differences of complete coding sequences within the monophyletic clusters detected in the Helsinki area ranged from 15 to 33 nucleotides (translated to 2–8 amino acids) in Espoo and 8–12 nucleotides (2–4 amino acids) in Sipoo. The molecular clock analyses based on complete coding sequences suggested estimated time to the most recent common ancestor (tMRCA) between 40.6 and 48.2 years [95% HPD range 20.5–76.8] for Espoo cluster and 36.9–42.2 years [95% HPD range 20.6–65.6] for Sipoo cluster, depending on the molecular clock model used (Table 2). The estimated tMRCAs for the Espoo/Kotka/Bornholm cluster were between 254 and 280 years [95% HPD 163-418].

**Table 2.** The tMRCAs and mean rates of evolution (substitutions/site/year) estimated using two clock models and two demographic models.

	Strict clock				Log normal relaxed clock			
	Constant population		Bayesian skyline		Constant population		Bayesian skyline	
	tMRCA	95% HPD	tMRCA	95% HPD	tMRCA	95% HPD	tMRCA	95% HPD
Espoo	48.2	23.9–76.8	43.6	22.4–65.2	45.3	22.4–73.3	40.6	20.5–62.5
Sipoo	42.2	22.0–65.6	38.8	21.6–58.6	39.7	21.2–62.4	36.9	20.6–56.7
Espoo/Bornholm/Kuutsalo	280.4	162.3–421.0	276.8	172.5–398	258.1	151.6–400.6	253.6	148.4–369
Mean rate	2.0476E-5	1.1958–2.9353E-5	2.0436E-5	1.3012–2.8399E-5	2.2377E-5	1.2916–3.3221E-5	2.2466E-5	1.3782–3.1689E-5
Co-efficient of variation					0.0927	7.7115–16.6E-3	0.0994	1.65–0.17.72E-3

## Discussion

The epidemiology of TBE has changed during the last decade in Finland [10]. In the Helsinki area, the capital area of Finland by the Gulf of Finland in the Baltic Sea, TBEV had been known to be endemic on only one outer island, Isosaari, since the 1990s [9], but no other observations of local TBEV transmission were made prior to the year 2011. In this study, we detected an increasing trend of TBE incidence and found evidence for local transmission and previously undetected endemic foci of TBEV in the Helsinki area.

Finland is at the northernmost limit of the TBE endemic area in Europe and here TBEV occurs in endemic foci. Since 2008, new foci have appeared annually in Finland [4,10–12,37,38]. Apart from the focus of Isosaari Island, no TBEV was recognized in the Helsinki area prior to 2011 when the first locally acquired human cases acquired in the Helsinki area were reported. In this study, we report an increased incidence of TBE in the residents of the Helsinki area. This increase was observed for both locally acquired and total reported cases. The increase in TBE incidence in the Helsinki area coincides with that of the whole Finnish population, in which the incidence has increased from 0.72to 1.11 between the years 2012–2016 (APC 14.4% [CI 0.7–28.1]) [46].

TBEV circulation in nature is very sensitive to various environmental factors such as microclimate and host animal population density [21,47–49]. Intriguingly, the sequence variation within the two foci of which more than one strain was sequenced was rather high, suggesting that the introduction of TBEV into these two foci has occurred most likely decades ago. According to climate models, the microclimate of the Helsinki area has been suitable for TBEV circulation already at the turn of the millennium [50]. It appears to have taken decades before introductions of TBEV into the area, ecological factors, and human contacts with the infected ticks all coincided in space and time for TBE to manifest as locally transmitted and notified cases from foci within the Helsinki area.

Further, the phylogenetic analysis indicated that the TBEV strains sequenced from the Sipoo archipelago, Espoo and Helsinki (Jollas peninsula and Isosaari Island) do not cluster together, although they are located only 20 km apart. This suggests independent introductions of four TBEV strains to the region. The strains from Espoo, on the other hand, are distantly related to the TBEV strain found in Kuutsalo, Kotka (140 km apart) [38]. However, the estimated tMRCAs for the Espoo/Kotka cluster were 250–280 years. With the current dataset, it cannot be concluded if these represent two independent introductions into Finland, or one introduction followed by a spreading of the virus along the coastline. Generally, the independent introduction of distinct TBEV lineages to a restricted geographical region is consistent with the studies conducted in Central and Southern Europe, where several TBEV lineages can be found in a given geographical region [40,44,51,52]. Thus, TBEV typically displays a high level of focus-specific clustering, but a low level of clustering within larger geographical areas. Most likely, however, the circulation of TBEV has been previously more limited in Finland and Scandinavia compared to Central Europe and the Baltic states.

Our results, together with suggestions of long distance dispersal events based on molecular epidemiology studies of TBEV [37,53] and findings of TBEV in ticks carried by migratory birds [54–58], are in line with the hypothesis that the initial introduction of TBEV has occurred via migratory birds, and followed with a subsequent local expansion of the established foci.

While all strains characterized from the Helsinki region were of the European subtype, both European and Siberian subtypes are known to circulate in Finland. Further, both virus subtypes have been found in both *Ixodes ricinus* and I. persulcatus vector species [4,11]. A previous study based on a large collection of ticks in 2015 demonstrated that only *Ixodes ricinus* was present in the southern coast of Finland, including the Helsinki area [5]. Tick collections not targeted at the suspected TBEV foci found TBEV prevalence in *I. ricinus* to be 0.2% [5]. Compared to this, the prevalences in the suspected foci reported in this study were high. TBEV prevalence was found to be 5.6% in ticks collected in Sipoo in the garden of a TBEV-infected patient. Also, one out of 35 mammals was TBEV RNA positive and six out of 35 mammals TBEV antibody positive in the Jollas peninsula of Helsinki, where three human cases of TBE have been recorded. Out of 655 ticks collected from various locations in Espoo, thirteen TBEV RNA positive ticks were found from sites where human cases have been reported, and only one from elsewhere. These results are consistent with the well-known very focal distribution of TBEV in nature. Even within the Helsinki area, TBE human cases, TBEV infection markers in wildlife samples and the presence of TBEV RNA in ticks seem to concentrate to only a few foci.

This study provides a description of the early events of establishment and subsequent spread of TBE foci in an urban area, as evidenced by diverse data including patient interviews, environmental sampling, TBEV screening, full-genome sequencing and sequence analysis. The findings are in concordance with observations on the changing epidemiology of TBE in the Nordic countries and suggest the further potential for TBEV emergence. Our results therefore call for further monitoring of the infection pressure, using One Health approaches to inform public health measures such as risk assessment and vaccine recommendations.
